# Substantial vertebral body osteophytes protect against severe vertebral fractures in compression

**DOI:** 10.1371/journal.pone.0186779

**Published:** 2017-10-24

**Authors:** Eric Wagnac, Carl-Éric Aubin, Kathia Chaumoître, Jean-Marc Mac-Thiong, Anne-Laure Ménard, Yvan Petit, Anaïs Garo, Pierre-Jean Arnoux

**Affiliations:** 1 Department of Mechanical Engineering, École de technologie supérieure, Montréal, Québec, Canada; 2 Research Center, Sacré-Cœur Hospital, Montreal, Quebec, Canada; 3 iLAB-Spine, Associated International Laboratory on Spine Biomechanics and Imagery, Montreal, Canada; 4 Department of Mechanical Engineering, École Polytechnique de Montréal, Montreal, Canada; 5 Research Center, Sainte-Justine University Hospital Center, Montreal, Quebec, Canada; 6 Department of medical imaging, North Hospital, Aix Marseille Université, Marseille, France; 7 Laboratoire d’Anthropologie Biologique, Aix Marseille Université, Marseille, France; 8 Department of Surgery, Faculty of medicine, Université de Montréal, Montreal, Quebec, Canada; 9 Laboratoire de Biomécanique Appliquée, IFSTTAR Aix Marseille Université, Marseille, France; 10 iLAB-Spine, Associated International Laboratory on Spine Biomechanics and Imagery, Marseille, France; University of Zaragoza, SPAIN

## Abstract

Recent findings suggest that vertebral osteophytes increase the resistance of the spine to compression. However, the role of vertebral osteophytes on the biomechanical response of the spine under fast dynamic compression, up to failure, is unclear. Seventeen human spine specimens composed of three vertebrae (from T5-T7 to T11-L1) and their surrounding soft tissues were harvested from nine cadavers, aged 77 to 92 years. Specimens were imaged using quantitative computer tomography (QCT) for medical observation, classification of the intervertebral disc degeneration (Thomson grade) and measurement of the vertebral trabecular density (VTD), height and cross-sectional area. Specimens were divided into two groups (with (n = 9) or without (n = 8) substantial vertebral body osteophytes) and compressed axially at a dynamic displacement rate of 1 m/s, up to failure. Normalized force-displacement curves, videos and QCT images allowed characterizing failure parameters (force, displacement and energy at failure) and fracture patterns. Results were analyzed using chi-squared tests for sampling distributions and linear regression for correlations between VTD and failure parameters. Specimens with substantial vertebral body osteophytes present higher stiffness (2.7 times on average) and force at failure (1.8 times on average) than other segments. The presence of osteophytes significantly influences the location, pattern and type of fracture. VTD was a good predictor of the dynamic force and energy at failure for specimens without substantial osteophytes. This study also showed that vertebral body osteophytes provide a protective mechanism to the underlying vertebra against severe compression fractures.

## Introduction

Vertebral body osteophyte is a common form of osteoarthritis defined as an abnormal bony growth or bone spur that forms along intervertebral joints [1]. In late stages of osteophytes development, adjacent vertebrae are fused together, thereby forming a bone bridge across the intervertebral disc called a bridging osteophyte. Vertebral osteophytes are generally associated with aging, intervertebral disc degeneration, traumatic injury, endplate sclerosis, disc space narrowing [1, 2], facet joint remodelling [3], back pain [4] and diffuse idiopathic skeletal hyperostosis (DISH) or Forestier disease [5]. Accordingly, a substantial osteophyte can be found in 20–25% of spines aged 20–45 years and in 73–90% of spines aged over 60 years [2–4, 6]. Complications associated with osteophyte formation are numerous (dysphagia, thoracic aortic compression, vena cava obstruction, nerve root compression) and affect most structures located in close-proximity to the spine. As a consequence, they are generally viewed as a degenerative condition and can be removed surgically by traditional or minimally invasive techniques during spinal surgery, when they lead to a disability or neurological symptoms [1, 7]. Recently, it was suggested that vertebral osteophytes primarily stabilize the spine in bending and increase its resistance in compression [8, 9]. Indeed, if osteophytes provide stability by adding bone and increasing the cross-sectional area of the vertebrae, they might also provide a protective mechanism against vertebral fractures despite the generalized osteopenia observed in most aging spines.

However, the mechanical role of vertebral body osteophytes concerning the risk and severity of vertebral fractures within the spine remains unclear. Most vertebral fractures range from painful compression fractures to more severe injuries such as burst and distraction fractures. Many of them are located at the mid-thoracic, thoracolumbar or mid-lumbar levels and result from an axial compression mechanism, with or without combined flexion [10]. To provide a better understanding of the biomechanics of vertebral fractures, many experimental studies have been conducted on human cadaveric tissues in axial compression. The load carrying capacity of individual vertebrae and functional spinal units (FSU) has formed the basis for quantifying the tolerance of the intervertebral structure to resist loading and remain unscathed [11–18]. Other complementary studies aimed to predict the risk of spinal fractures by identifying potential correlations between the failure load/vertebral fractures of individual vertebrae or FSU and clinical measurements such as the bone mineral density (BMD) and morphology [19–24], the degree of degeneration [25] and the bone architecture [26–28].

Although the aforementioned experiments have provided significant insight into the biomechanics of vertebral fractures, none of them have explained the mechanical contribution of vertebral osteophytes. Recently, it was suggested that vertebral osteophytes increase the resistance of the spine to compression and that in their presence, clinical BMD measurements underestimate vertebral compressive strength [9, 29]. However, their cadaveric tests were performed under physiological loads (no failure) applied quasi-statically. Hence, they did not replicate the dynamic bona fide conditions of trauma situations (e.g. falls or traffic accidents) that lead to vertebral compression fractures.

The present study aimed to analyze the role of substantial vertebral osteophytes on the biomechanical response of the spine under dynamic compression, up to failure. Their influence on the prediction of vertebral compressive strength by clinical measurements was also investigated. Our hypothesis is that substantial vertebral osteophytes have a significant influence on failure parameters, type, pattern, location and risk prediction of vertebral fractures when the spine is submitted to dynamic compression.

## Materials and methods

### Cadaveric specimens

The research protocol was approved by the institutional review board of IFSTTAR/Aix-Marseille University. None of transplant donors were from a vulnerable population and all donors or next of kin provided written informed consent that was freely given. Seventeen human spine segment specimens composed of three-vertebrae and their surrounding soft tissues were harvested from nine embalmed cadavers. The average age, standing height and weight of the subjects were 86 ± 5 years, 164 ± 6 cm and 60 ± 13 kg (Table 1). Seven specimens were located at T11-L1, seven at T8-T10, and three at T5-T7. All bony ribs and non-ligamentous soft tissues were removed from the specimens and quantitative computed tomography (QCT) images were taken using a clinical scanner (SIEMENS Somatom Sensation Cardiac 64: 120 kV, 300–350 mAs, contiguous slices of 0.6 mm thickness) for medical observation, classification and measurement of the osteophytes, which were observed at all vertebral levels. Each specimen was classified according to the direction of each pair of osteophytes across the intervertebral disc space [30] and according to the Thomson’s disc degeneration grading scheme [31]. The size of the osteophytes was measured using the method described by Al-Rawahi et al [9]. Specimens were divided into two groups (Table 1). The first group (n = 9), named ‘specimens with substantial osteophytes’, had type C osteophytes, characterized by an almost complete bone bridge formed across the intervertebral disc space. Their osteophytes ranged from 10 to 15 mm. The second group (n = 8), named ‘specimens with small osteophytes’, had type E osteophytes, characterized by osteophytes that extended nearly horizontally to the vertebral body border without closing the intervertebral disc space. Their osteophytes ranged from 2 mm (which is the minimum size to consider a spur an osteophyte [32]) to 5 mm.

**Table 1:** Details of the 17 spinal specimens extracted from the 9 cadavers.

Subject ID	Gender	Age (years)	Weight (kg)	Standing height (cm)	Specimen	Group	IVD grade
01	F	88	64	155	T11-L1	1	5
02	F	83	49	165	T8-T10	2	3
03	F	85	48	154	T11-L1	1	5
04	F	92	54	164	T8-T10	1	5
					T11-L1	2	4
05	F	93	39	165	T8-T10	2	3
					T11-L1	2	3
06	F	88	71	165	T8-T10	2	4
					T11-L1	2	4
07	M	77	75	173	T5-T7	2	3
					T8-T10	1	3
					T11-L1	2	3
08	M	90	91	170	T5-T7	1	5
					T8-T10	1	5
					T11-L1	1	5
09	M	82	53	165	T5-T7	1	5
					T8-T10	1	5

Measurements of the vertebral trabecular density (VTD), the height (H) and the cross-sectional area (CSA) of each specimen were then performed. The VTD was measured on each vertebra by creating a 9 mm thick scanning section parallel to the vertebral endplates, at the mid plane of the vertebral body [33]. The mid-vertebral body location was confirmed by the entry of the basi-vertebral vein on the mid-plane of the scanning section. The mean Hounsfield unit (HU) was computed over an oval region of interest of 25 mm^2^ that included trabecular bone only (no cortical rim or parts of the basi-vertebral vein). For standardization (calibration) purposes, mean HU values of each vertebra were converted into VTD values (g/mm^3^), by using linear regression derived from a CBCT Electron Density Phantom (Model 062A, CIRS Inc., Virginia, USA) placed next to the spine specimens during QCT. The specimen’s VTD was finally computed by averaging the VTD of its three composing vertebrae. The height of the specimen was measured between the center of the superior and inferior endplates of the caudal and distal vertebrae, respectively. The CSA of each vertebra was measured at the middle of their vertebral body. The global CSA of the specimen was obtained by averaging the CSA of its three vertebrae. After imaging, specimens were frozen at -20°C until testing.

### Testing apparatus and procedures

**Fig 1:**
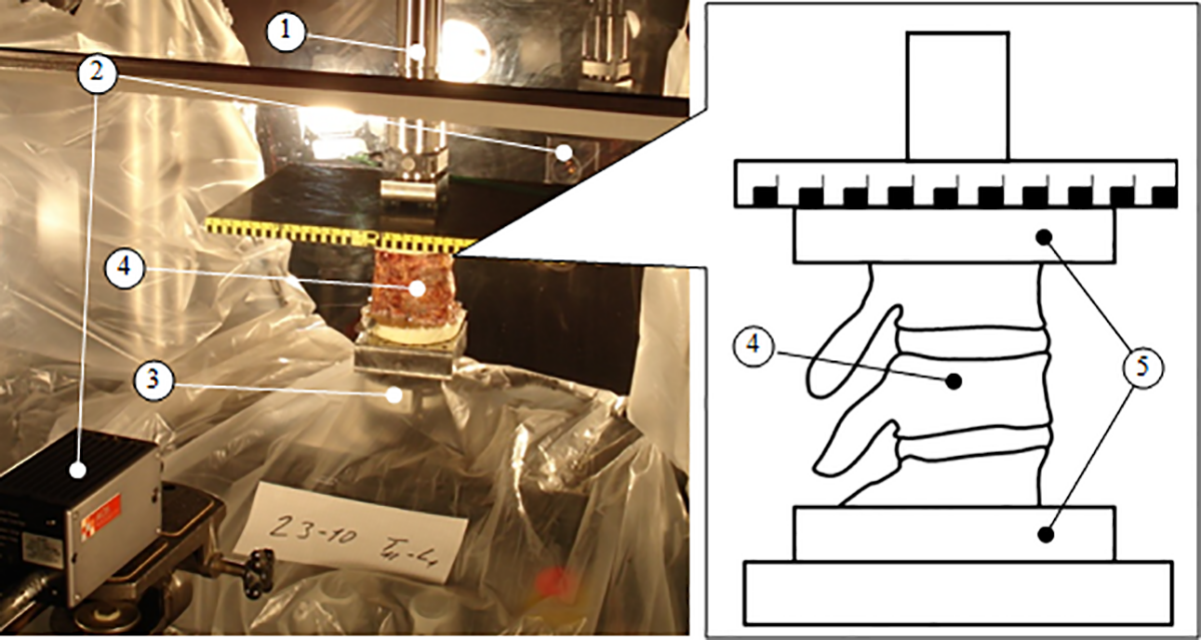
Experimental setup composed of a servohydraulic Material Testing System (1), two high-speed cameras (2), a single-unit load cell (3), and a cadaveric spinal segment (4). The cadaveric spinal segment (4) was fixed on the MTS by potting the distal and proximal vertebrae in polyurethane resin (5).

Dynamic axial compression was performed using a servohydraulic Material Testing System (MTS Axial 370.02 15 kN, Eden Prairie, MN) that included a single axis load cell and an inductive displacement transducer to respectively measure the actuator force and deflection (Fig 1). Two high-speed cameras (1000 frames/second) were placed aside of the specimen to visualize and chronologically trace spinal lesions. Two days (48 h) prior to testing, specimens were thawed in a refrigerator (4°C) and covered by saline soaked towels. To fix the specimen on the MTS and load it uniformly, half of the distal and proximal vertebrae were potted in parallel cups filled with a polyurethane resin twenty-four hours before testing. During this process, the sagittal natural curvature of the specimens was maintained.

Prior to the test, specimens were thawed at room temperature (23°C) for 3 hours. Each specimen was positioned on the MTS and a 0.25 kN preload was applied for 1 minute [20]. Specimens were then dynamically compressed at a displacement rate of 1 m/s, up to failure. The test was stopped when the specimen was compressed to approximately 60% of the height of its middle vertebra (12 to 15 mm). All tests were performed at room temperature. Following compression, QCT images of the specimens were taken for medical diagnosis.

### Data analysis and statistics

Force-displacement curves were recorded and normalized (F_norm_ = f(D_norm_)) according to the mean size of all specimens, as described by Eqs (1) and (2):

$$F_{norm}=\frac{F}{\frac{CSA_{specimen}}{MeanCSA}}=F.\frac{MeanCSA}{CSA_{specimen}}$$ (1)

$$D_{norm}=\frac{D}{\frac{Height_{specimen}}{MeanHeight}}=D.\frac{MeanHeight}{Height_{specimen}}$$ (2)

This normalization process was performed to pool together stiffness and failure parameters from different genders, subject sizes and spinal levels. Failure was defined as the first point where a further increase in displacement caused a decrease in load. Normalized stiffness (*K*), force (*F*_*FAIL*_), displacement (*D*_*FAIL*_) and energy at failure (*E*_*FAIL*_) were extracted from each curve. Stiffness was defined as the slope of the most linear portion of the curve, prior to failure. Failure energy was defined as the area under the force-displacement curve, up to failure. The mean and standard deviations of the biomechanical parameters (*K*, *F*_*FAIL*_, *D*_*FAIL*_ *and E*_*FAIL*_) were computed for each group and statistically compared. Replication of the tests and pure estimates of the error variance for the biomechanical parameters could not be performed since the tests were destructive.

Ordinary linear regression models were fitted to estimate the amount of linear correlation between VTD and each measured parameter for each group, without considering any interaction between the parameter. To check whether the relationship between VTD and the normalized biomechanical parameters for the group with small osteophytes was similar to the group with substantial osteophytes, the model fitted to the former group was used to predict the biomechanical parameters of the other group. The biomechanical parameters (mean and standard deviation) calculated from these relationships were then compared to the biomechanical parameters obtained experimentally for the other group.

Videos were analyzed to identify failure location and fracture patterns. QCT images of the fractured specimens were used to identify the type of fracture based on the AO (initials for the German terms ‘Arbeitsgemeinschaft für Osteosynthesefragen’) classification for thoracic and lumbar injuries [34]. Chi-squared tests were performed to investigate the influence of the osteophytes on the location, type and pattern of fracture. All statistical analyses were performed using STATISTICA v.7.0 (StatSoft, Tulsa Oklahoma, USA). Differences were considered significant at p < 0.05.

## Results

### Morphology and vertebral trabecular density

Specimens with substantial osteophytes showed higher (1.6 times) VTD than specimens with small osteophytes (p = 0.003) (Table 2, S1 Fig and S1 Table). They also showed higher cross-sectional area (p = 0.307), but lower height (p = 0.071). However, as opposed to the VTD, differences in the gross morphology of the specimens were not significant.

**Table 2:** Vertebral trabecular density (VTD), cross-sectional area (CSA) and specimen height (H) by group of specimens (mean ± standard deviation).

	Specimens with substantial osteophytes (n = 9)	Specimens with small osteophytes (n = 8)	Ratio (substantial/small)
VTD (g/mm^3^)	103 ± 24	64 ± 25	1.60*
CSA (mm^2^)	1062 ± 171	1009 ± 251	1.05
H (mm)	77.0 ± 5.0	81.3 ± 6.6	0.95

* significant difference between groups (p = 0.003).

### Force-displacement curves

**Fig 2:**
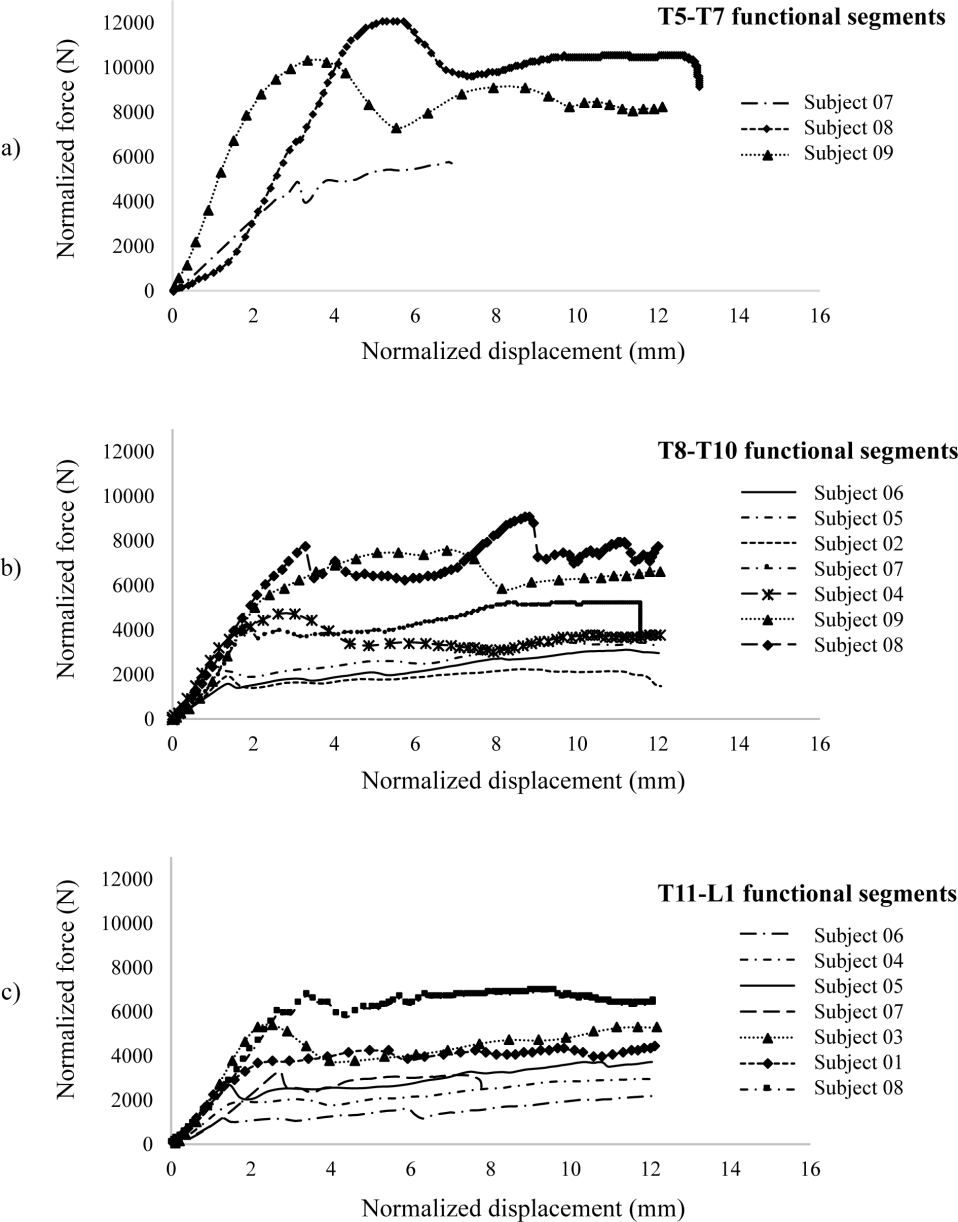
Normalized force-displacement curves for the T5-T7 (a), T8-T10 (b) and T11-L1 (c) specimens. Curves with symbols represent specimens with substantial osteophytes. These specimens showed greater stiffness, loads and displacements before failure.

**Fig 3:**
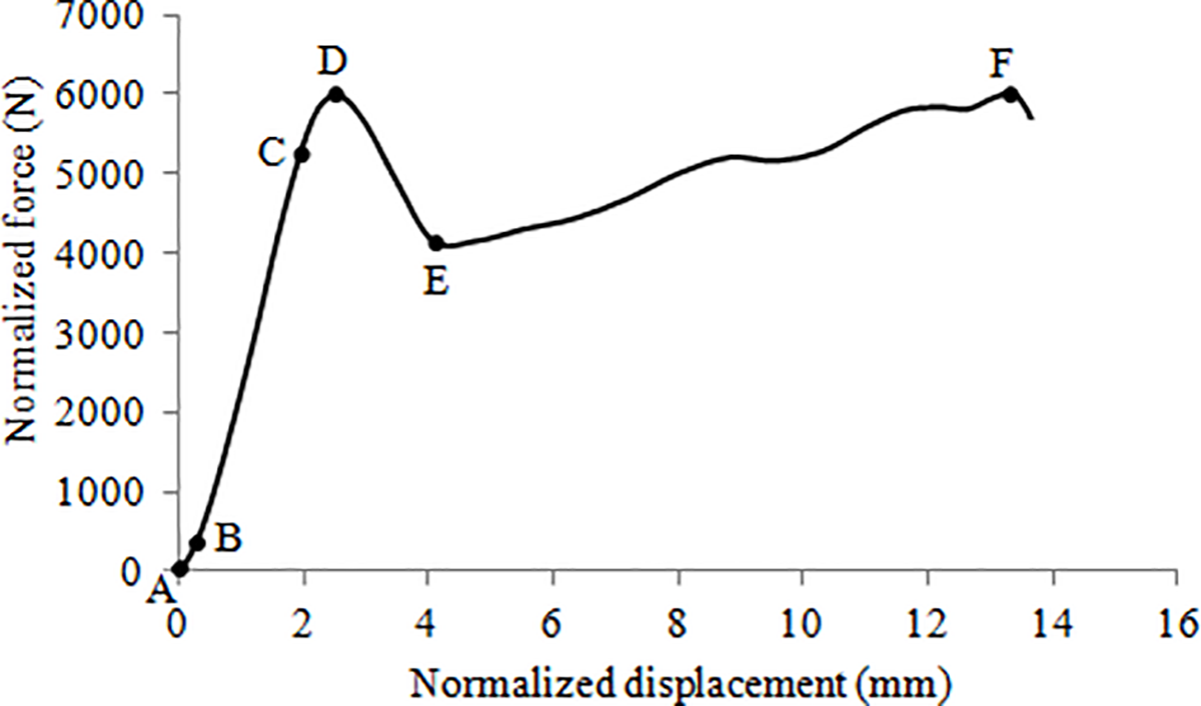
Typical force-displacement (F-D) curve (specimen T11-L1, subject 3). The six segments (from A-B to E-F) depicted on this curve were observed on all specimens.

All specimens exhibited normalized force-displacement curves of similar shapes (Fig 2). Curves with embedded markers (squares, triangles, crosses, etc.) represent specimens with substantial osteophytes. A typical curve is depicted in Fig 3. Prior to failure, the curve exhibits a non-linear shape characterized by a first curved segment of increasing stiffness (segment A-B), immediately followed by a linear segment (B-C). This linear segment precedes a highly non-linear segment (C-D) characterized by a decreasing stiffness. Segment B-C is referred to as the elastic portion of the curve while segment C-D is referred to as the inelastic portion where micro-level damage (loss of material continuity via formation of cracks and voids which degrade stiffness), plasticity (flow process that creates irrecoverable strains) and viscoelasticity (dissipative process) take place. After failure (point D), the curve exhibits a decreasing strength (D-E) up to point E, after which the specimen either maintains (nine specimens) or regains (eight specimens) some strength (segment E-F).

### Normalized stiffness and failure parameters

The average normalized stiffness (*K*) of specimens with substantial osteophytes was 1.9 times the stiffness of specimens with small osteophytes (Table 3). The average *F*_*FAIL*_, *D*_*FAIL*_ and *E*_*FAIL*_ of specimens with substantial osteophytes were 2.7, 1.8 and 5.3 times the corresponding values of specimens with small osteophytes, respectively. All differences between groups were statistically significant, with p-values of 0.0004 (*K*), 0.0006 (*F*_*FAIL*_), 0.006 (*D*_*FAIL*_), and 0.002 (*E*_*FAIL*_).

**Table 3:** Normalized stiffness and failure parameters by group (mean ± standard deviation).

	Specimens with substantial osteophytes (n = 9)	Specimens with small osteophytes (n = 8)		Ratio (substantial/small)
K (N/mm)	3207 ± 735	1716 ± 586		1.9†
F_FAIL_(N)	6873 ± 2534	2510 ± 1152		2.7†
D_FAIL_ (mm)	3.5 ± 1.1	1.9 ± 0.9		1.8†
E_FAIL_ (J)	14.8 ± 8.1	2.8 ± 2.4		5.3†

† significant difference between groups (*K*: p = 0.0004; *F*_*FAIL*_: p = 0.0006; *D*_*FAIL*_: p = 0.006; *E*_*FAIL*_: p = 0.002).

### Correlations between normalized biomechanical parameters and VTD

**Fig 4:**
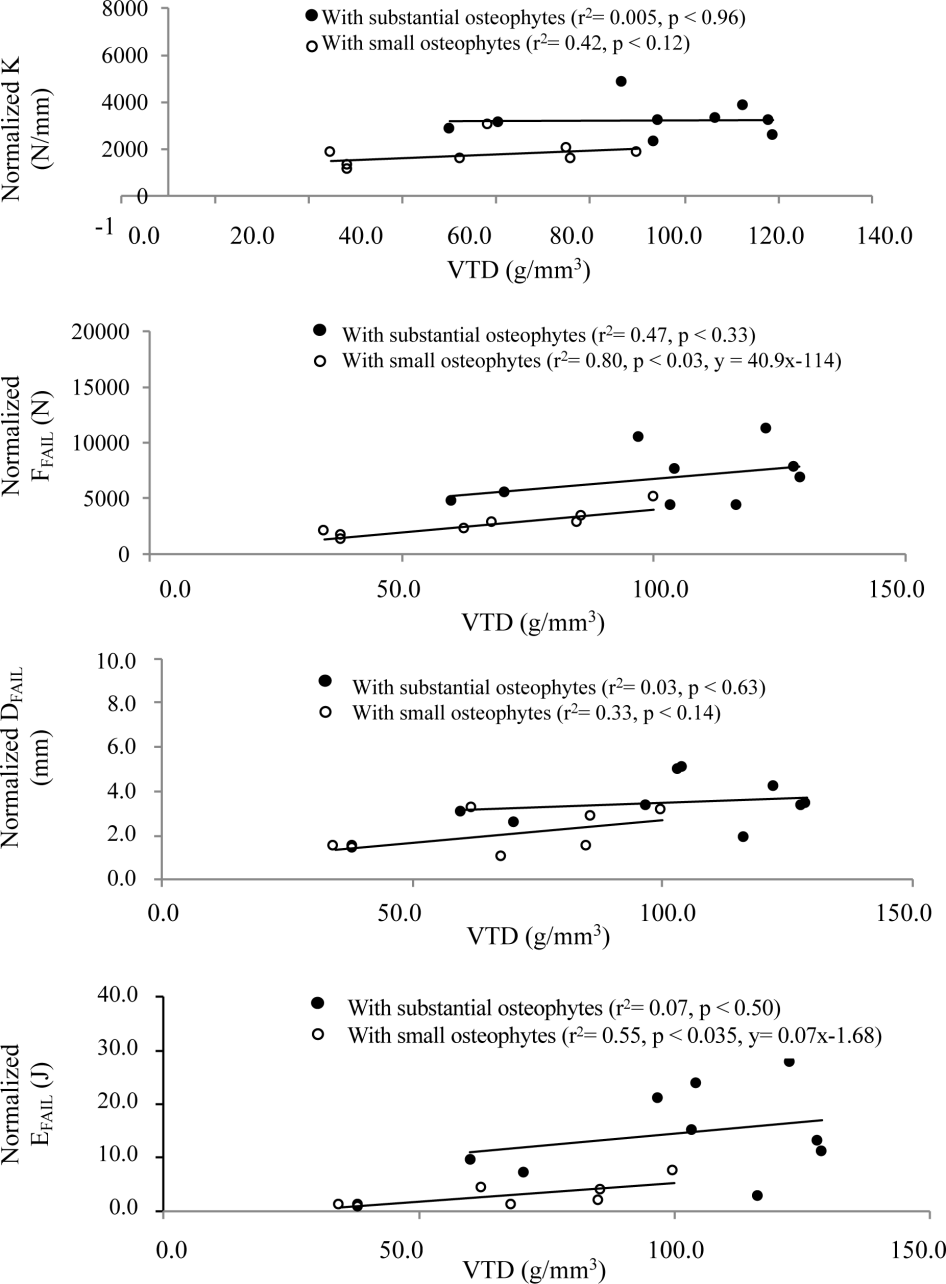
Linear regressions between the VTD and the normalized biomechanical parameters. The VTD was only correlated with normalized *F*_*FAIL*_ and *E*_*FAIL*_ of specimens without osteophytes.

For specimens with substantial osteophytes, no statistically significant correlation was found between the VTD and the biomechanical parameters (Fig 4). For specimens with small osteophytes, the VTD was a good predictor of the normalized *F*_*FAIL*_ (r^2^ = 0.80, p < 0.03) and *E*_*FAIL*_ (r^2^ = 0.55, p < 0.035). In contrast, the VTD was not correlated with *K* (r^2^ = 0.42, p < 0.12) and *D*_*FAIL*_ (r^2^ = 0.33, p < 0.14).

Following these results, values for *F*_*FAIL*_ and *E*_*FAIL*_ were predicted using the linear relations of Fig 4 and the VTD of the specimens with substantial osteophytes, so that specimens with similar VTD could be compared. Again, significant differences were found between groups (*F*_*FAIL*_ = 4117 ± 1002 N; *E*_*FAIL*_ = 5.5 ± 1.7), thus ensuring that VTD alone could not explain the differences observed between groups.

### Fracture patterns, types and locations

**Fig 5:**
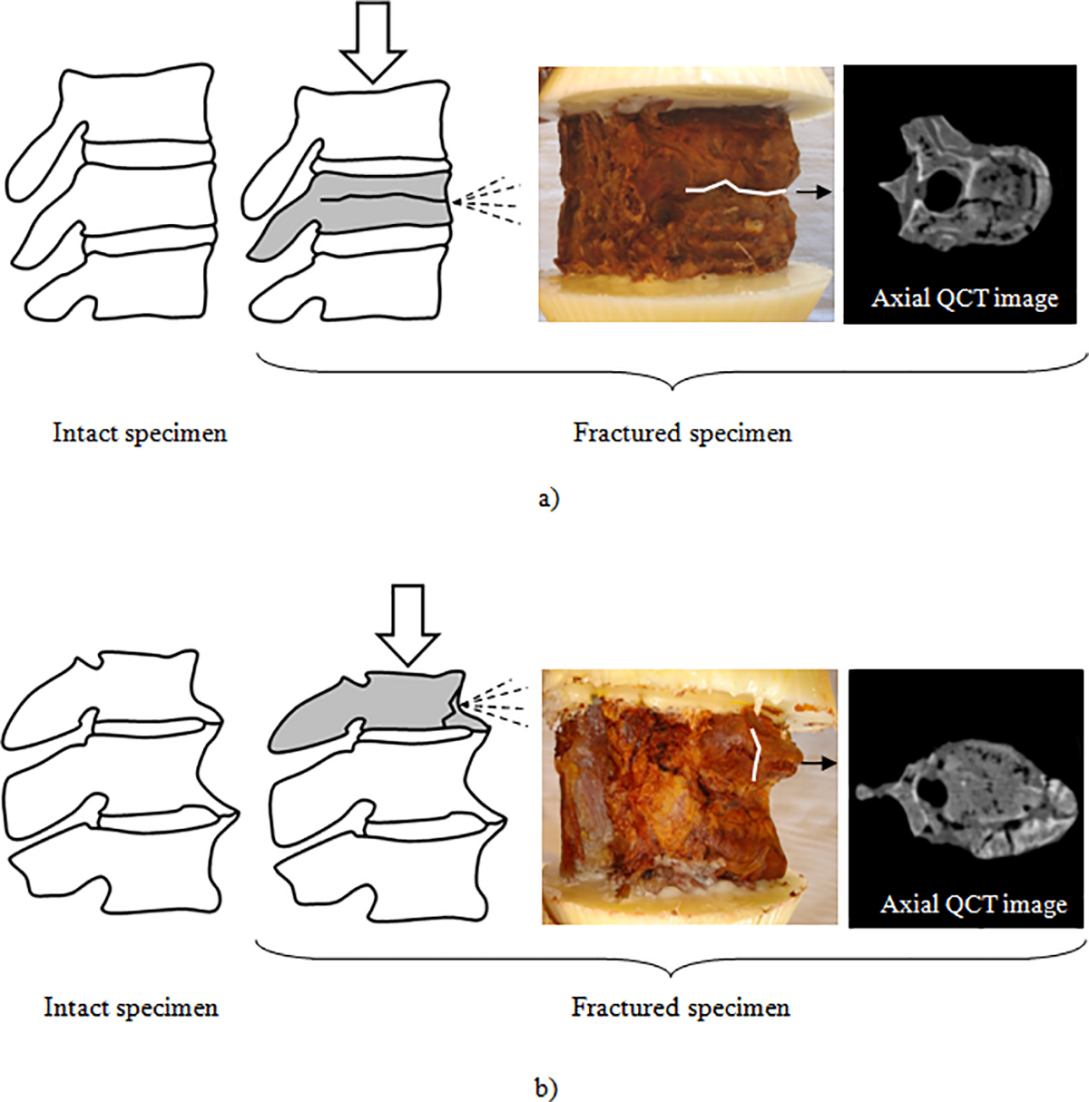
First vertebra to collapse and fracture pattern. a) Specimen without osteophytes (segment T11-L1 specimen of subject 3). The middle vertebra (T12) is the first to collapse. A horizontal pattern splits the vertebra in upper and lower parts. A burst fracture is observed on the QCT image. b) Specimen with substantial osteophytes (segment T8-T10 of subject 2). The proximal vertebra (T11) is the first to collapse. A vertical pattern splits the vertebra in anterior and posterior parts. A fracture of the osteophyte is observed on the QCT image. The dashed lines represent the ejection of the bone marrow at the site of the fracture.

At failure, video analyses showed that one of the vertebrae collapsed and bone marrow was expulsed in jet streams at the site of fracture, and from the orifices and vascular channels in the cortex. The first vertebral body to collapse and the fracture pattern were significantly different between the two groups of specimens. Indeed, the first vertebral body to collapse was the middle or distal one for seven of the eight specimens with small osteophytes (Fig 5A), while it was the proximal one for eight of the nine specimens with substantial osteophytes (Fig 5B).

Three patterns of fracture were identified on the cortical surfaces. In the first pattern, the cortical bone splinters circumferentially and horizontally at its center, leaving a line that separates the vertebral body in upper and lower parts (Fig 5A). In the second pattern, the cortical bone splinters vertically, leaving a line that mostly divides the vertebral body in anterior and posterior portions (Fig 5B). The third pattern was a combination of the two other patterns and resulted in a complete destruction of the specimen. The first pattern was observed on seven of the eight specimens with small osteophytes, while the second pattern was observed on seven of the nine specimens with substantial osteophytes. The third pattern was observed on one specimen of each group.

QCT images revealed that the presence of substantial osteophytes had an influence on the types of fractures (Table 4). Five of the specimens with small osteophytes sustained a complete burst fracture with a retropulsed bone fragment and canal compromise (type A.3.3 of the AO classification) while three of these specimens sustained an impaction fracture with collapse of the vertebral body (type A.1.3). Only one specimen with vertebral osteophytes had a complete burst fracture (with fracture of the osteophyte). The other eight specimens had an impaction fracture with vertebral body collapse (type A.1.3), sometimes with a small displacement of the posterior cortical wall. Amongst these specimens, six had fractures of at least one of their osteophytes.

**Table 4:** Distribution of the types of fracture by group of specimens.

Types of fracture	Specimens with substantial osteophytes (n = 9)	Specimens with small osteophytes (n = 8)
Burst fracture		
- Alone	0	5
- With fracture of an osteophyte	1	0
Impaction fracture or vertebral body collapse		
- Alone	2	3
- With fracture of an osteophyte	6	0

## Discussion

Previous investigations [9, 35] showed that vertebral osteophytes induce significant changes in the resistance and flexibility of FSU under quasi-static or physiological loading conditions. The present study distinguishes itself as longer spinal segments (three-vertebrae) under dynamic compression were tested and the role of vertebral body osteophytes on fracture mechanisms of spinal segments assessed. Substantial vertebral osteophytes increase the stiffness and load-bearing capacity of spinal segments. They also influence the pattern, location and risk-prediction of vertebral fractures.

The limitations of the present study must be discussed before interpreting the findings. Firstly, because of the limited availability and cost of human cadavers, only seventeen specimens were extracted from nine subjects. This small sample size is an important limitation of the study. Therefore, to maximize sample sizes, data from different genders and spinal levels were pooled together using a normalization process. However, anthropometric differences between males and females (Table 1) could have reduced or masked potential correlations with VTD, although both groups contained male and female samples. It would be interesting to study the effect of gender and find gender-specific correlations with more samples. In this perspective, biomechanical parameter individual data was made available for further use (S1 Fig and S1 Table). Also, vertebral body sizes depend on gender; therefore, accounting for vertebral cross sections is of the utmost importance in the expression of the force to compare samples. The normalization process, similar to the generation of engineering stress-strain curves, was selected so that biomechanical parameters were expressed in units consistent with those commonly used by clinicians or reported in other studies [9, 16, 36, 37]. Normalized force was preferred to compressive stress to express loading as vertebrae are non-homogeneous and complex materials. Normalization was based on the average mid-vertebral body CSA and the total height of the specimens since these morphologic parameters were unaltered by the presence of osteophytes. Secondly, conservation conditions of the specimens could have influenced the results, but freezing and storage do not significantly alter their physical properties [38–40]. Finally, the applied loading and boundary conditions were simplified in comparison to real-life trauma situations (falls, car crashes, etc.) due to very limited knowledge on the complex loads involved in such situations and to the inherent difficulty of replicating these loads experimentally. However, the resulting fractures were similar to those observed clinically and the energy applied to the specimens was in good agreement with the energy applied in other *in vitro* experiments aiming to replicate compression fractures [41–44].

Both groups of specimens showed consistent mechanical responses up to failure, with normalized force-displacement curves showing similar shapes (segments A-B to C-D) as force-displacement curves reported in other studies [16, 37]. Moreover, the main failure forces of 6873 ± 2534 N and 2510 ± 1152 N respectively observed on specimens with substantial and small osteophytes were in the same range as those reported in similar experiments performed on healthy lumbar spinal segments of two and six vertebrae [37]. After failure, both groups of specimens were able to maintain or increase their strength (segments E-F). This characteristic could be explained by the crushing mechanism of the cancellous bone. Lindahl [45] observed similar curve patterns after compressing small cubic blocks of lumbar trabecular bone. Carter and Hayes [46] suggested that as the load increases, the collapse of an increasing number of intertrabecular spaces constrains the bone marrow movement, thus providing a hydraulic cushion to the spinal segment. They further reported that specimens with increasing strength following failure were associated with a higher overall bone mineral density. In the current study, specimens with vertebral osteophytes were associated with a higher VTD and were able to sustain greater loads after failure (Fig 2). At this point, it is not possible to confirm whether this higher VTD, which was measured within the cortical rim, can be attributed to osteophyte formation or to biological variations between specimens. In a further study, analyzing specimens using fracture patterns of a larger sample size could also improve correlations with VTD.

The biomechanical parameters of specimens with small osteophytes were in good agreement with those reported in other experimental studies performed in similar conditions [36, 37, 47]. The higher *K* and *F*_*FAIL*_ measured for specimens with substantial osteophytes agreed well with the results of Al-Rawahi et al. [9], who showed on FSU that osteophytes multiply these parameters by factors of 1.17 and 1.18, respectively. Our higher multiplication factors (1.9 and 2.7 respectively) might be attributed to the different loading conditions (dynamic vs quasi-static compression) and the nature and size of the tested osteophytes (the length of the osteophytes in Al-Rawahi et al. [9] averaged 7 mm and none of them showed a complete and rigid anterior bridge of bone). This latest observation seems relevant since increasing severity of osteophyte formation is related to concomitant vertebral disc space narrowing [2], which in turn induces stiffening of the spinal segment in compression [35]. Regression analysis showed that the significant differences between groups for *F*_*FAIL*_ and *E*_*FAIL*_ could not be solely explained by the significant difference in VTD, thus confirming the role of vertebral osteophytes on the failure behaviour of spinal specimens.

For specimens with small osteophytes, the strong linear relation between the VTD of the specimen and *F*_*FAIL*_ and *E*_*FAIL*_ is in good agreement with results obtained in quasi-static conditions [20, 24] and is convincing since the failure force is a property that usually depends on density. In contrast, none of the failure parameters for specimens with substantial osteophytes was correlated with VTD, suggesting that substantial osteophytes produce significant bony alterations that add uncertain contributions to vertebral strength. Similar conclusions were reported by Al-Rawahi et al. [9] with BMD measurements from dual photon x-ray absorptiometry. This finding suggests that the clinical evaluation of the risk of fractures for specimens with substantial osteophytes should not only rely on the VTD and the gross morphology of the specimen until further studies are provided.

By supporting most of the compressive load until failure, substantial vertebral osteophytes seem to provide stress-shielding of the underlying vertebral body and to prevent the occurrence of a complete transverse and horizontal disruption of the vertebral body (often associated with burst fracture). The high occurrence of burst fracture in specimens with small osteophytes, despite a mildly or severely degenerated disc (grade 3 or 4), could be attributed to the higher shear forces transmitted by the facets to the posterior wall of the vertebra. Moreover, the protective mechanism provided by the vertebral osteophytes could be attributed to their ability to modify the resistance to deformation of adjacent discs (by increasing their CSA) or to their higher bone density. These findings support the hypothesis that osteophytes are adaptive rather than degenerative [9], and that their presence allows reducing excessive bone strains to normal levels.

In the present study, cadaveric tests were performed to assess the role of vertebral body osteophytes on the biomechanical response of spinal segments under dynamic compression. Reported results confirm our hypothesis that substantial vertebral osteophytes play a key-role in the failure parameters, type, pattern, location and risk prediction of vertebral fractures. Future works would benefit from the use of computational models [48–50] to circumvent the inherent limitations related to *in vitro* tests (sample size, biological variations, simplified loads, etc.). In this respect, data provided in the current study might be helpful to develop and validate these tools.

## Supporting information

S1 Fig Individual data for each subject ordered by group with substantial osteophytes and with small osteophytes. (TIF)

S1 Table Individual data for each subject: Vertebral trabecular density (VTD), cross-sectional area (CSA), specimen height (H), stiffness (*K*), and failure parameters (*F*_*FAIL*_, *D*_*FAIL*_, and *E*_*FAIL*_) without normalization. (DOCX)
