# Supplementary figures and images for: Substantial vertebral body osteophytes protect against severe vertebral fractures in compression
Source: PLoS One. 2017 Oct 24;12(10):e0186779. doi: 10.1371/journal.pone.0186779 (PMC5655488; doi:10.1371/journal.pone.0186779)

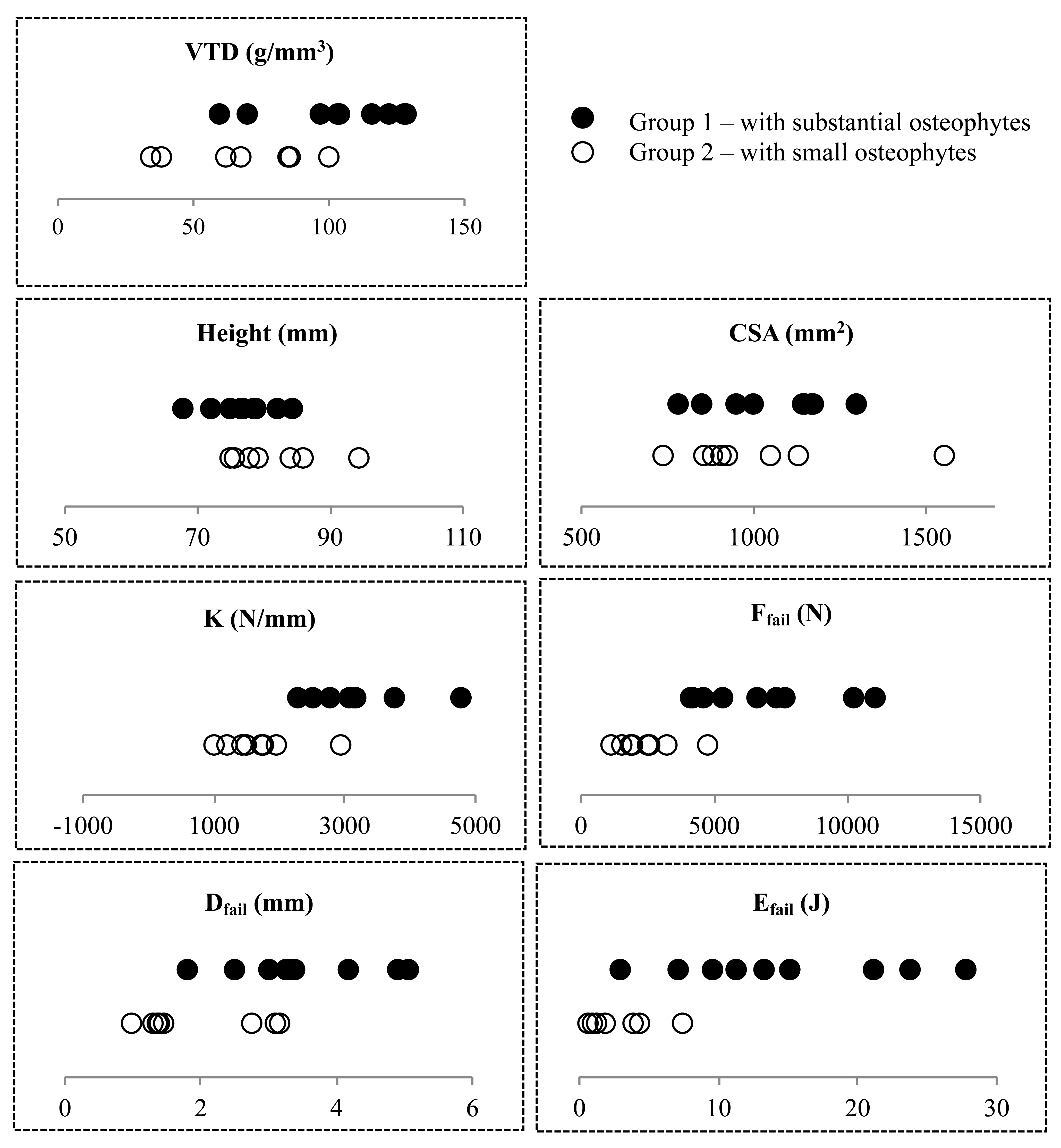

Supplement: S1 Fig — (TIF) [file pone.0186779.s001.tif]
