# Supplementary material for: Substantial vertebral body osteophytes protect against severe vertebral fractures in compression
Source: PLoS One. 2017 Oct 24;12(10):e0186779. doi: 10.1371/journal.pone.0186779 (PMC5655488; doi:10.1371/journal.pone.0186779)
Supplement: S1 Table — (DOCX) [file pone.0186779.s002.docx]

S1 Table. Individual data for each subject: vertebral trabecular density (VTD), cross-sectional area (CSA), specimen height (H), stiffness (*K*), and failure parameters (*F_FAIL_*, *D_FAIL_*, and *E_FAIL_*) without normalization.

| Subject | Vertebral level | Group | VTD  (g/mm^3^) | CSA (mm^2^) | Height (mm) | K (N/mm) | F_FAIL_ (N) | D_FAIL_ (mm) | E_FAIL_ (J) |
| --- | --- | --- | --- | --- | --- | --- | --- | --- | --- |
| 1 | T11-L1 | 1 | 103,2 | 1172,5 | 68,0 | 2301 | 4244 | 4,92 | 15,20 |
| 2 | T8-T10 | 2 | 34,7 | 929,9 | 75,7 | 1788 | 1923 | 1,39 | 1,30 |
| 3 | T11-L1 | 1 | 70,4 | 1145,3 | 82,3 | 3114 | 5413 | 2,50 | 7,20 |
| 4 | T8-T10 | 1 | 60,0 | 956,3 | 76,7 | 2796 | 4705 | 3,02 | 9,70 |
|  | T11-L1 | 2 | 62,4 | 1054,4 | 86,2 | 1460 | 2034 | 3,17 | 4,50 |
| 5 | T8-T10 | 2 | 68,1 | 741,3 | 78,0 | 2955 | 2560 | 0,98 | 1,30 |
|  | T11-L1 | 2 | 85,1 | 859,8 | 84,0 | 1981 | 2680 | 1,46 | 1,90 |
| 6 | T8-T10 | 2 | 38,3 | 883,7 | 75,0 | 1245 | 1577 | 1,38 | 1,10 |
|  | T11-L1 | 2 | 38,3 | 1131,7 | 79,3 | 1032 | 1178 | 1,30 | 0,70 |
| 7 | T5-T7 | 2 | 100,0 | 912,0 | 77,9 | 1744 | 4843 | 3,11 | 7,50 |
|  | T8-T10 | 1 | 116,4 | 1179,7 | 78,9 | 3210 | 4226 | 1,81 | 3,10 |
|  | T11-L1 | 2 | 85,9 | 1555,7 | 94,6 | 1523 | 3282 | 2,76 | 3,90 |
| 8 | T5-T7 | 1 | 122,5 | 856,0 | 76,9 | 3782 | 11086 | 4,18 | 27,90 |
|  | T8-T10 | 1 | 127,8 | 1004,1 | 78,6 | 3157 | 7719 | 3,27 | 13,40 |
|  | T11-L1 | 1 | 128,9 | 1302,3 | 84,4 | 2531 | 6718 | 3,40 | 11,40 |
| 9 | T5-T7 | 1 | 96,8 | 782,7 | 72,0 | 4800 | 10310 | 3,35 | 21,20 |
|  | T8-T10 | 1 | 104,3 | 1155,3 | 74,9 | 3175 | 7434 | 5,05 | 23,80 |
